# The sweetest thing: the influence of angularity, symmetry, and the number of elements on shape-valence and shape-taste matches

**DOI:** 10.3389/fpsyg.2015.01382

**Published:** 2015-09-15

**Authors:** Alejandro Salgado-Montejo, Jorge A. Alvarado, Carlos Velasco, Carlos J. Salgado, Kendra Hasse, Charles Spence

**Affiliations:** ^1^Crossmodal Research Laboratory, Department of Experimental Psychology, University of OxfordOxford, UK; ^2^Escuela Internacional de Ciencias Económicas y Administrativas, Universidad de La SabanaChía, Colombia; ^3^Department of Industrial Engineering, Pontificia Universidad JaverianaBogotá, Colombia; ^4^Imagineering InstituteIskandar, Malaysia

**Keywords:** crossmodal correspondences, shapes, taste words, aesthetics, emotional valence

## Abstract

A within-participants experiment was conducted in two countries (the UK and Colombia) in order to investigate the matching of shapes to taste words. Comparing the two countries allowed us to explore some of the cultural differences that have been reported thus far solely in terms of people's visual preferences. In particular, we addressed the question of whether properties other than angularity influence shape-valence and shape-taste matching (crossmodal correspondences). The participants in the present study repeatedly matched eight shapes, varying in terms of their angularity, symmetry, and number of elements to one of two words—pleasant or unpleasant and sweet or sour. Participants' choices, as well as the latency of their responses, and their hand movements, were evaluated. The participants were more likely to judge those shapes that were rounder, symmetrical, and those shapes that had fewer elements as both pleasant and sweet. Those shapes that were more angular, asymmetrical, and that had a greater number of elements, were more likely to be judged as both unpleasant and sour instead. The evidence presented here therefore suggests that aside from angularity and roundness, both symmetry/asymmetry and the number of elements present in a shape also influence valence and taste categorizations.

## Introduction

Research on the crossmodal correspondences (also known as synesthetic correspondences, Martino and Marks, 2001), underscores the existence of linguistic (or semantic), statistical, and structural relations to explain how/why seemingly unrelated information from different senses is matched to one another (see Spence, 2011; Parise and Spence, 2013; Deroy and Spence, 2015, for reviews). However, none of the aforementioned mechanisms would really seem to provide an adequate account for the crossmodal matches that have been documented recently between shapes and basic tastes (e.g., Velasco et al., 2015a).

Emotional valence (see Kenneth, 1923, for an early example; see also Collier, 1996; Lyman, 1979) would seem to provide one plausible mechanism that may help to explain shape-taste correspondences (Spence and Deroy, 2013a). Emotional valence can be understood as the assessment of whether a situation/object is perceived as helpful/harmful or rewarding/threatening (see Lane et al., 1999; Feldman, 2006). Velasco et al. (2015a) recently demonstrated that taste hedonics (as assessed by liking ratings) were correlated with the crossmodal associations that people made between tastes and the roundness/angularity of shapes. These researchers also suggested that other shape attributes might be involved in shape-taste matches as well (see also Wan et al., 2014; Velasco et al., 2015b,c). Furthermore, given that crossmodal correspondences are thought to be bidirectional (see Spence, 2011; Deroy et al., 2013; Parise and Spence, 2013), the suggestion is that both taste and shape properties may influence shape-taste matching.

Köhler's (1929) seminal early work in which participants were asked say the words “Bouba” and “Kiki” and to match them to a rounder or a more angular shape, revealed that people do indeed match sounds to shapes. Köhler's results further suggested that shapes could be matched to information in other modalities. In the intervening years, there has been a growing interest in understanding how shapes are matched to tastes^1^. However, shape taste-matching research has tended to focus primarily on the influence of one polar shape property; roundness/angularity (e.g., Maurer et al., 2006; Spence and Gallace, 2011; Ngo et al., 2013, but see also Cytowic and Wood, 1982; and see Spence and Deroy, 2013a, for a review), leaving aside other shape aesthetic properties that may also be relevant to explaining shape-taste matches. Here, think only of stimulus attributes such as symmetry and the number of elements that a shape possesses. Evidence from the fields of psychology (Berlyne, 1960, p. 244, 1970; Reber et al., 2004), neuroscience (see Chatterjee, 2011, for a review), evolutionary biology (Enquist and Arak, 1994; Enquist and Johnstone, 1997), and art theory (Collingwood, 1963; Hobbs and Salome, 1991), demonstrates that different visual properties influence the perceived valence of a stimulus. Several studies have, for example, demonstrated that symmetry is associated with a positive valence (e.g., Makin et al., 2012). According to aesthetics theory, the number of elements that a shape possesses can also influence people's expressed preference, with shapes having fewer elements being preferred over those with more (e.g., Jacobsen et al., 2006). Nevertheless, subsequent attempts to confirm this claim have tended to yield mixed findings (see Palmer et al., 2013, for a review on visual aesthetics and preference).

The evidence that has been published to date would appear to suggest that roundness/angularity is a prominent feature when it comes to associating shapes with tastes. Nevertheless, the question remains as to whether the shapes that are currently used to study shape-taste matches necessarily constitute the best representation, or whether other features (such as, for example, symmetry) also influence such crossmodal correspondences. The idea here is that the valence of a shape is determined by its various features, and taste/shape correspondences are influenced by the emotional valence of the component stimuli. That said, it is possible to hypothesize that the manipulation of such features may influence the correspondence between taste and shape.

What is more, cross-cultural differences have also been reported between Western and non-Western populations (e.g., Bremner et al., 2013; see also Wan et al., 2014). For example, Bremner et al. reported that members of the Himba tribe in Namibia matched carbonation to rounder shapes, whereas Westerners seem to match it to more angular shapes. These differences could perhaps be explained (at least in part) by disparities in people's aesthetic judgments and preferences toward specific visual properties (e.g., roundness/angularity, symmetry, and for number of elements, see Tinio and Leder, 2009; Jacobsen, 2010). Further research is therefore needed in order to address the question of whether different cultures have distinct shape-taste and valence-shape matches.

As with the case of shapes, different tastes have also been associated with a specific valence. For instance, sweet and umami are usually associated with approach states, whereas sour and bitter tastes signal substances that may potentially be dangerous (e.g., rotten meat or overripe fruits and vegetables, and poisons). Our response to salty tastes, on the other hand, seems to depend on our physiological needs (see Yarmolinsky et al., 2009, for a review). Given that sweetness and sourness have previously been associated with shapes and have also been linked to pleasurable and dangerous substances, respectively, these two tastes provide good candidates to study how different shape properties influence shape-taste correspondences.

The present study has two main objectives: First, we wanted to determine whether characteristics such as roundness/angularity, symmetry/asymmetry, and the number of elements that go into making-up a particular shape can influence the crossmodal correspondences that are observed between shapes and tastes. Second, we wanted to evaluate whether the aforementioned characteristics would also be associated with a specific emotional valence (e.g., unpleasant/pleasant). We were also interested in determining whether shape-valence and shape-taste matching are similar in two different countries, namely the United Kingdom and Colombia. Here, it is important to note that different studies have reported cultural differences in the valence attributed to different visual features (e.g., such as the number of elements, see Masuda et al., 2008; Tinio and Leder, 2009; Jacobsen, 2010). Such results therefore, emphasize the need to explore how different countries match specific shape properties with an emotional valence and whether this influences the shape-taste matches that they exhibit. Moreover, it is also interesting to study the effects so far reported in countries that have received less attention from researchers, as this would certainly help to strengthen any generalization derived from experimental research (see Henrich et al., 2010).

**Figure 1:**
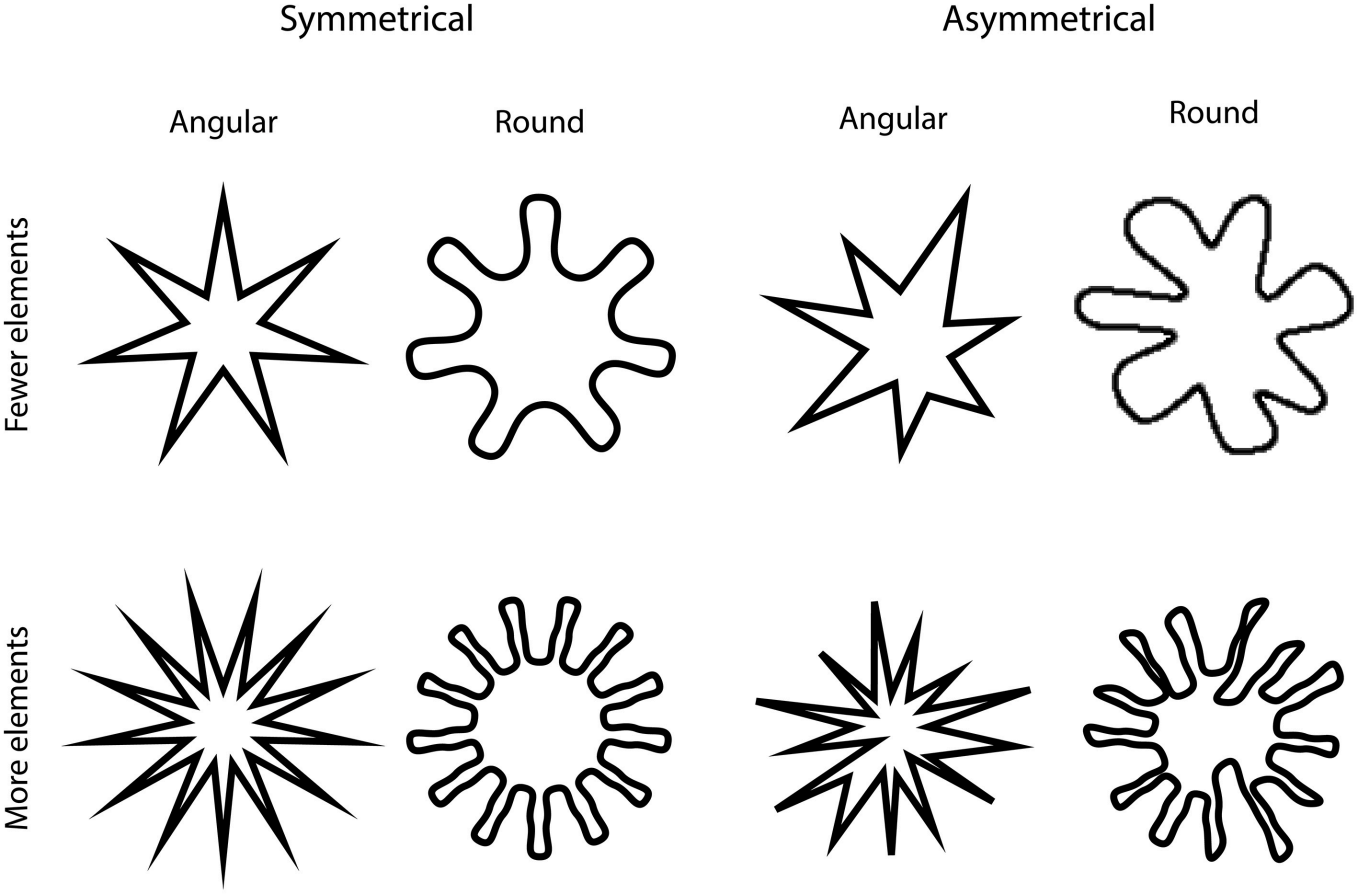
**The visual stimuli that were presented to the participants in the two tasks**.

The participants in the present study had to match eight shapes in two forced choice tasks. The order of the two tasks was counterbalanced across participants. In one of the tasks, the participants had to match the shapes to the words “pleasant” or “unpleasant,” in the other task, they matched the same shapes to the words “sweet” or “sour” instead. Symmetry/asymmetry, roundedness/angularity, and the number of elements in each shape were varied (see Figure 1). Shape-taste matches were evaluated using an experimental paradigm that has not been used before in this context, namely, mouse tracking (Tipper et al., 1992; Papesh and Goldinger, 2012; Yu et al., 2012). As suggested by Tipper et al. registering responses via keypresses may fail to account for how some real-world interactions take place. By presenting participants with tasks where they need to move their hand toward the response and tracking hand movements it may sometimes be possible to better understand how decision processes evolve.

Our hope was that the results of these studies would be able to show that emotional valence is involved in the associations that participants make between shapes and tastes. We also expected that roundness, symmetry, and a smaller number of elements would be matched with a positive valence (i.e., pleasant) and the word sweet. By contrast, those shapes that are angular, asymmetrical, and those shapes possessing a greater number of elements would be matched to a negative valence (i.e., unpleasant) and to the word sour instead. We anticipated that our participants' performance (i.e., reaction times—RTs—and hand movements), would be influenced by the different shape properties. In particular, those shapes that are matched with a negative valence and to the word sour were expected to yield faster RTs than those shapes matched with a positive valence and to the word sweet. We also expected that shapes that presented properties that conveyed conflicting valences or tastes would generate slower RTs and more hand movements (reflecting uncertainty, or conflict) on the part of the participant. Our hope in conducting the present research was partly that the findings could potentially be applied to the development of brands, product packaging, and advertising that can more consistently/effectively communicate a specific the taste using visual cues (see Salgado-Montejo et al., 2014; Velasco et al., 2014; Ghoshal et al., 2015).

## Methods

### UK participants

Twenty-six participants (15 male, mean age = 29.05 years, *SD* = 9.03, ranging from 18 to 55), recruited via the database of the Crossmodal Research Laboratory at the University of Oxford took, part in the study. The experiment was reviewed and approved by the Central University Research Ethics Committee at the University of Oxford (MS-IDREC-C1-2014-056). All of the participants had normal or corrected-to-normal vision. The participants signed a standard consent form at the start of their session and were compensated with £5 for taking part in the study.

### Colombian participants

Thirty-seven participants (18 female, mean age = 20.7 years, *SD* = 2.29, ranging from 18 to 28) were invited to take part in the study via the database of the International School of Economics and Administrative Science at Universidad de La Sabana in Colombia. The experiment was reviewed and approved by the Research Committee of the International School of Economics and Administrative Science at Universidad de La Sabana. The participants were compensated with academic credit for taking part.

### Apparatus and materials

The participants sat at approximately 50 cm. in front of a 14″ LED monitor, with a screen resolution of 1024 × 768 pixels, and a screen refresh rate of 60 Hz. Adobe Illustrator CS6 was used to create eight different shapes (see Figure 1). The stimuli were developed based on the shapes first introduced by Köhler (1929) and later by Ramachandran and Hubbard (2001). The original shapes presented by Köhler (see shapes on the top right corner of Figure 1) were taken as a reference point and they were modified by making them symmetrical or by increasing the number of elements that make-up each shape. The different shapes were presented to the participants using MouseTracker Software, Version 2.82. (Freeman and Ambady, 2010). For the Colombian sample, all words in the experiment were presented in Spanish (Sweet, Dulce; Sour, Ácido; Pleasant, Agradable; Unpleasant, Desagradable).

### Procedure

Two 2 × 2 × 2 experimental designs with factors shape roundness/angularity, shape symmetry/asymmetry, and the number of elements (7 vs. 12), were conducted. The participants were informed that they would be presented with different shapes (see Figure 1) and two words (i.e., sweet and sour or pleasant and unpleasant) located at the top-left and top-right of the screen.

**Figure 2:**
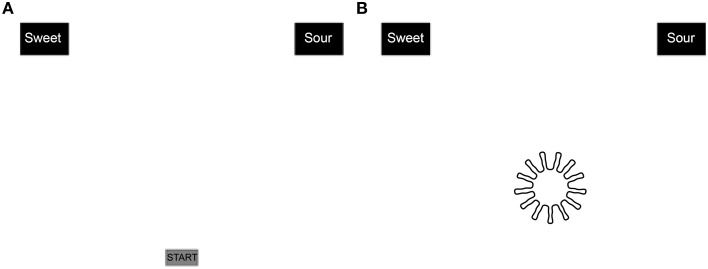
**Schematic visualization of the task presented to participants showing the screen before (A) and after (B) the participants had clicked the start button on the screen**.

The experiment comprised two tasks; in each task, the participants were first presented with written instructions on the screen. They then had to decide which of two words best matched the shape presented on the screen. During one of these tasks, they had to match each shape to either the words “pleasant” or “unpleasant.” In the other task, the participants had to match the shapes to the words “sweet” or “sour” (in Spanish) instead. The order of presentation of the two tasks was counterbalanced across participants. In both tasks, the shapes were presented on the lower center of the screen and afterwards the participants had to move the mouse and click on one of two possible words (see Figure 2). Each of the eight shapes was presented four times (32 trials) and the position of the words presented on the screen (either unpleasant and pleasant or sweet and sour) was counterbalanced.

### Analyses

A generalized estimating equations (GEE) method was used to analyze the data (see Liang and Zeger, 1986)^2^. Given the nature of the data from this experiment, a binary logistic regression GEE was used to analyze the choices that were made by the participants. RTs were analyzed with a scalar logistic regression GEE and, for the motor complexity indexes, a Poisson logistic regression GEE was used. All GEE used a hybrid estimation method and a marginal model. Participants' choices, as well as reaction times (RTs), and motor complexity indexes for hand movements were evaluated. Trials with RTs that were greater than two standard deviations from the mean were discarded from the analyses. The total number of trials did not go over 2% of the entire data set in either of the two samples.

## Results

**Figure 3:**
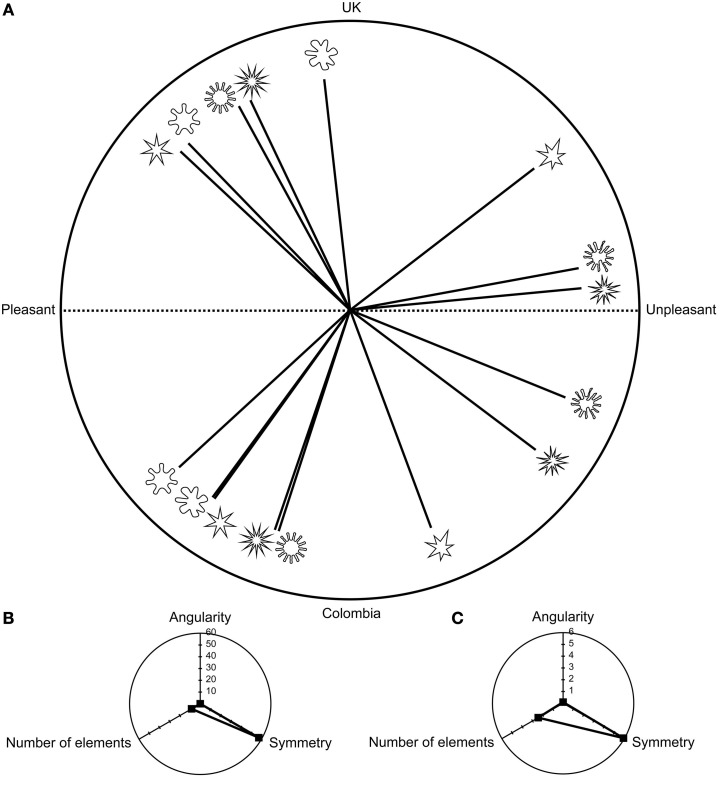
**Figures showing (A) the mean categorization frequencies for the valence task presented as angles in a 180° range for each of the shape properties**. The upper part of the circle presents the frequencies for the UK sample, while the bottom part of the circle presents the frequencies for the Colombian sample. Shapes that were more consistently rated as pleasant are presented toward the left, while those shapes that were deemed to be more unpleasant are presented toward the right. **(B)** The odds ratios for the UK sample representing the influence (i.e., effect size) that each shape property had on the categorizations made by the participants; and **(C)** The odds ratios for the Colombian sample. **(B,C)** show the overall effect of each shape property on the categorization of each in terms of a valence word. These panels help the reader to understand the order in which the shapes appear in **(A)**, and why there are some variations between countries. Note that scale for the effect sizes is different in the UK and Colombian sample.

An overview of the results revealed the same main effects in both countries (i.e., the UK and Colombia) in both tasks (i.e., shape-valence and shape-taste matching). It would appear that symmetry/asymmetry and the number of elements seemed to exert the greatest influence as to how the participants matched each shape to a valence word (See Table 1 and Figure 3). In contrast, in the taste task, roundness/angularity, symmetry/asymmetry, and the number of elements had varying degrees of influence, on the participants of each country, on how shapes were matched to a taste word (see **Table 3** and **Figure 6**). Note that we only present in Figures, and in the main text, those effects that displayed significant differences (see Appendix Table A1 for a report of the χ^2^ and *p*-values of all the effects that were tested).

**Table 1:** **The aggregated frequencies per each of the shape properties for the valence and taste tasks**.

**Property**	**Valence**		**Taste**	
	**Pleasant**	**Unpleasant**	**Sweet**	**Sour**
Round	529	479	634	374
Angular	451	557	183	825
Total	980	1036	817	1199
Symmetry	684	324	467	541
Asymmetry	296	712	350	658
Total	980	1036	817	1199
Less elements	610	398	558	450
More elements	370	638	259	749
Total	980	1036	817	1199

*A total of 2016 trials were recorded for each task*.

The results are presented in detail below as a function of task, measurement, and country.

### Pleasant/unpleasant task

The UK and Colombian samples displayed a significant main effect of symmetry/asymmetry [UK: Wald $\chi_{\left(1\right)}^{2}=54.51$, *p* < 0.001, Colombia: Wald $\chi_{\left(1\right)}^{2}=33.07$, *p* < 0.001] and the number of elements [UK: Wald $\chi_{\left(1\right)}^{2}=25.00$, *p* < 0.001, Colombia: Wald $\chi_{\left(1\right)}^{2}=37.26$, *p* < 0.001, see also Figure 3]. Symmetry was more likely to be matched to the word pleasant when compared with asymmetry (Odds ratio–OR–UK: 57.46; OR Colombia: 5.92). Those shapes having fewer elements also presented a higher likelihood of being matched to the word pleasant (OR UK: 8.47; OR Colombia: 2.44). Symmetry/asymmetry presented the highest influence on the likelihood of matching a shape to a specific valence in both countries. The shapes that were more easily matched to either the word pleasant or unpleasant varied between countries (see Figure 3A). These differences and the overall frequencies can be explained by the influence (i.e., effect sizes) of each of the shape properties on the likelihood of matching a specific shape to a valence word (see Figures 3B,C).

As shown by the interactions (see Table 2 and Figure 3), roundness/angularity seems to require the presence of other shape properties in order to communicate valence (i.e., symmetry/asymmetry in the UK and the number of elements for the Colombian sample). In both countries, arrangements of symmetry and number of elements that communicated the same valence (i.e., more symmetrical shapes and shapes with fewer elements or more asymmetrical and shapes with a greater number of elements) increased the odds of matching a particular shape with a valence word (OR UK: 4.77; OR Colombia: 1.61). For the Colombian sample, congruent roundness/angularity x symmetry/asymmetry × number of elements properties further increased the odds of matching a shape with a specific valence (OR UK: 2.19; OR Colombia: 4.84)

**Table 2:** **The influence of each shape property on shape-valence matches as shown by binary logistic regression GEE**.

**Shape properties**	**UK**	**Colombia**
Roundness/angularity	No effect	No effect
Symmetry/asymmetry	Symmetrical = pleasant^***^	Symmetrical = pleasant^***^
Number of elements	Fewer elements = pleasant^***^	Fewer elements = pleasant^***^
Roundness/angularity × symmetry	Rounder × symmetrical = pleasant^*^	No effect
Roundness/angularity × number of elements	No effect	Rounder × fewer elements = pleasant^**^
Symmetry/asymmetry × number of elements	Symmetrical × fewer elements = pleasant^***^	Symmetrical × fewer elements = pleasant^***^
Roundness/angularity × symmetry/asymmetry × number of elements	No effect	Rounder × symmetrical × fewer elements = pleasant^**^

* p < 0.05;

** p < 0.01;

*** *p < 0.001. Note that all effects that are significant include both polar properties (e.g., symmetry and asymmetry, or roundness and angularity)*.

### RTs

**Figure 4:**
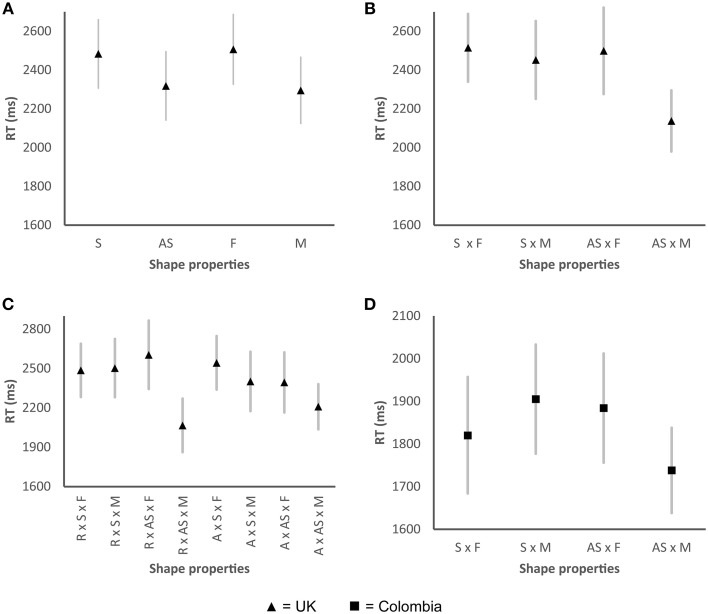
**Graphs showing the mean RTs for the valence task for those shape properties that displayed significant differences**. **(A)** Shape properties that displayed significant differences in the UK sample; **(B)** Significant two-way interaction between symmetry and the number of elements for the UK sample; **(C)** Significant three-way interaction between roundness/angularity, symmetry, and the number of elements for the UK sample; **(D)** Significant two-way interaction between symmetry/asymmetry and the number of elements in the Colombian sample. R, round; A, angular; S, symmetrical; AS, asymmetrical; F, fewer elements; M, more elements. Error bars, standard error.

A significant main effect of symmetry/asymmetry was found in the UK sample, Wald $\chi_{\left(1\right)}^{2}=8.45$, *p* < 0.01, as well as a main effect of the number of elements Wald $\chi_{\left(1\right)}^{2}=15.88$, *p* < 0.001 (see Figure 4A). The participants responded faster (Mean difference, MD: 166 ms, *p* < 0.01) to shapes that were asymmetrical (Confidence Interval, CI: 2142–2495 ms) as compared to those shapes that were symmetrical (CI: 2307–2660 ms, see also Figure 4A). Shapes that presented a greater number of elements (CI: 2124–2466 ms) were also categorized more rapidly (MD: 212 ms, *p* < 0.001) by the participants when compared to shapes with fewer elements (CI: 2327–2687 ms, see also Figure 4A). A significant interaction was found between symmetry/asymmetry x the number of elements Wald $\chi_{\left(1\right)}^{2}=8.66$, *p* < 0.01, with the participants responding even more rapidly to those shapes that were asymmetrical and presented a greater number of elements (see Figure 4B).

The participant responded more rapidly (MD: 314 ms, *p* < 0.001) to asymmetrical shapes that had a greater number of elements (CI: 1980–2295 ms) as compared to those shapes that had a greater number of elements but which were symmetrical (CI: 2251–2653 ms, see also Figure 4B). A significant three-way interaction was also found, with shapes that were rounder, asymmetrical, and which presented more elements generating faster RTs (see Figure 4C). The participants categorized the shapes that appeared to be matched with the word unpleasant (i.e., shapes with a greater number of elements and/or asymmetrical shapes) more quickly than those shapes that were matched to the word pleasant (i.e., those shapes with fewer elements and/or symmetrical shapes).

In the Colombian sample, RTs displayed a significant interaction between symmetry/asymmetry × number of elements Wald $\chi_{\left(1\right)}^{2}=12.45$, *p* < 0.001. The participants responded more rapidly (MD: 167 ms, *p* < 0.001) when presented with shapes that had more elements and were asymmetrical (CI: 1638–1838 ms) as compared to those shapes that were made-up of more elements and were symmetrical (CI: 1777–2033 ms, see Figure 4D). No other effects were found for either of the two samples.

### Motor complexity

**Figure 5:**
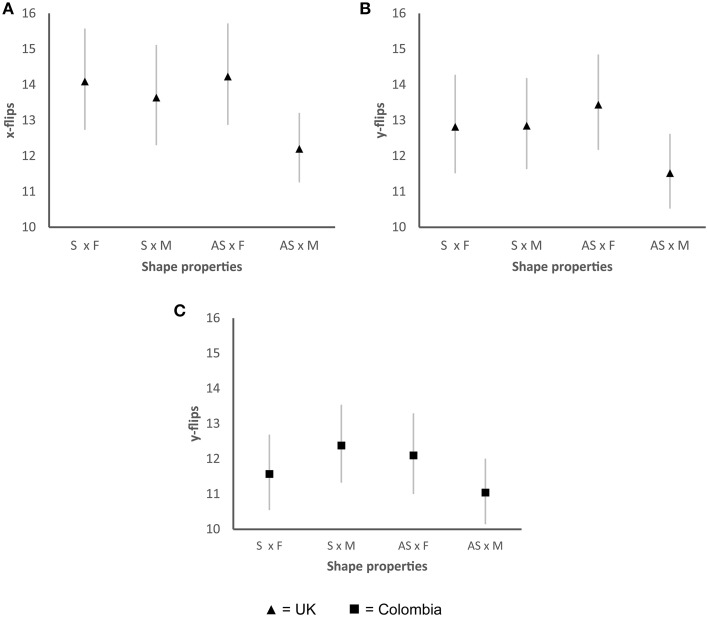
**Graphs showing the mean hand flips for the valence task for those shape properties that displayed significant interactions**. Main effects are not shown given that the interactions explain the main effects. **(A)** Significant two-way interactions between symmetry/asymmetry and the number of elements in the x-axis for the UK sample; **(B)** two-way interactions between symmetry/asymmetry and the number of elements in the y-axis for the UK sample; **(C)** two-way interactions between symmetry/asymmetry and the number of elements in the y-axis for the Colombian sample. Significant differences were found when comparing shapes that were both asymmetrical and had fewer elements with the rest of the conditions (AS × M). S, symmetrical; AS, asymmetrical; F, fewer elements; M, more elements. Error bars, standard error.

Motor complexity was calculated by counting the number of hand movements toward and away from the response (y-flips) and from left to right (x-flips) and creating an index to standardize these movements across participants. The motor complexity index is calculated by counting the number of hand reversals on each axis (x and y), before each participant clicked the mouse on each trial (see Freeman and Ambady, 2010). A significant main effect was found in the UK participants for the number of elements in the number of x-flips Wald $\chi_{\left(1\right)}^{2}=10.26$, *p* < 0.01 and y-flips Wald $\chi_{\left(1\right)}^{2}=6.03$, *p* < 0.01. A significant interaction was found for the UK sample between symmetry/asymmetry and the number of elements in the x-axis Wald $\chi_{\left(1\right)}^{2}=4.28$, *p* < 0.05 and in the y-axis Wald $\chi_{\left(1\right)}^{2}=8.68$, *p* < 0.01. There were less hand movements prior to making a response when the participants were presented with shapes that were asymmetrical or had more elements. Shapes that were both asymmetrical and presented more elements further reduced the participants' hand movements prior to clicking a valence word (see Figures 5A,B). No other effects were found for the UK sample. A significant interaction was observed between symmetry/asymmetry and the number of elements Wald $\chi_{\left(1\right)}^{2}=4.91$, *p* < 0.05 in the y-flips of Colombian participants. Again, the participants generated less hand movements when shapes were both asymmetrical and presented more elements (see Figure 5C). No other significant effects were found for the Colombian sample. Less flips in both axes suggest that the participants found it easier to associate a specific shape with a valence word. No other effects were found.

### Sweet/sour task

**Figure 6:**
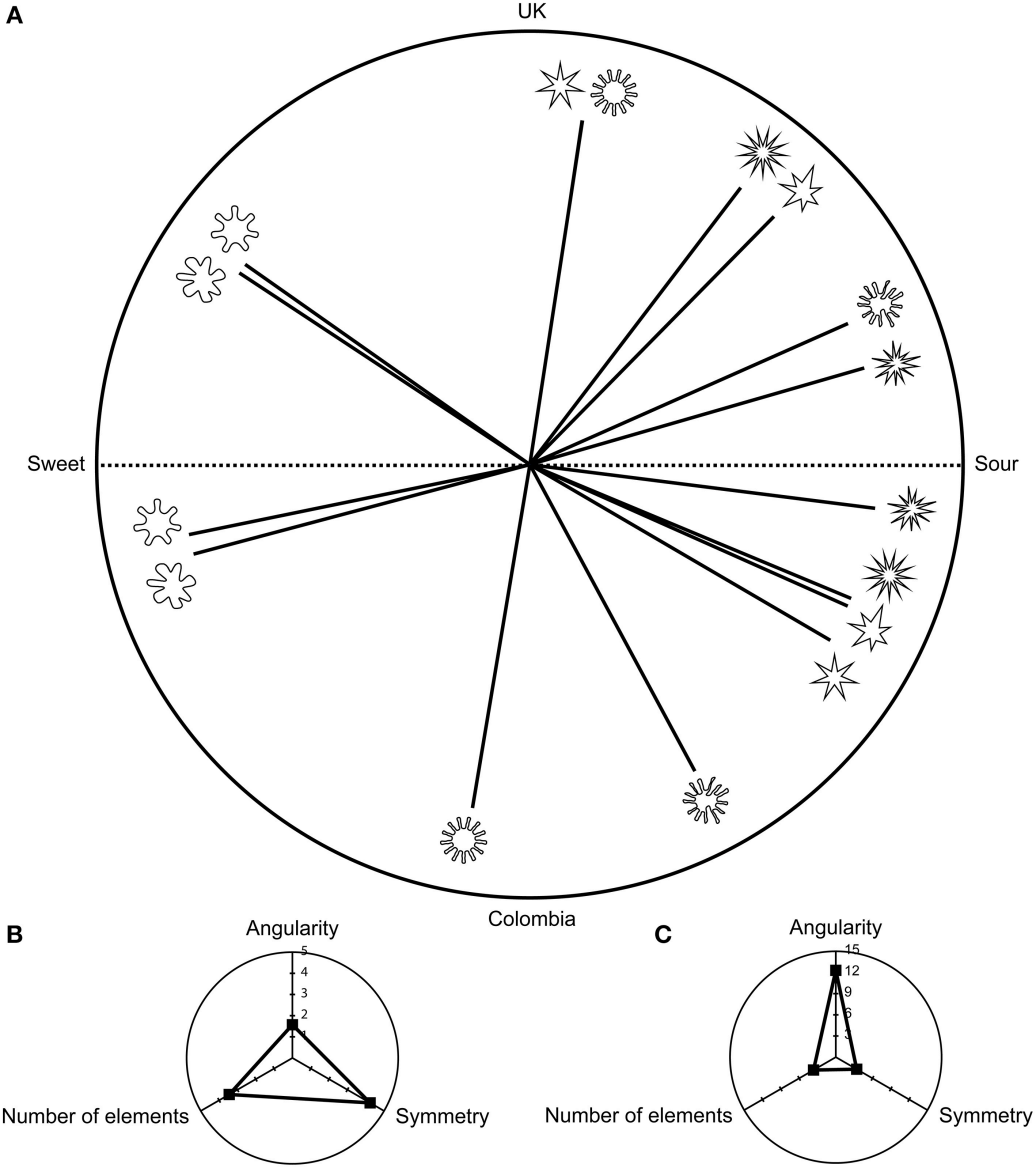
**Figures showing (A) the mean categorization frequencies for the taste task**. The upper part of the circle presents the frequencies for the UK sample, while the bottom part of the circle presents the frequencies for the Colombian sample. Shapes that were more consistently rated as sweet are presented toward the left, while those shapes that were deemed sour are presented toward the right. **(B)** The odds ratios for the UK; **(C)** The odds ratios for the Colombian sample. Given that **(B,C)** present the effect size for each shape property, they can be used as an explanatory measure to help understand the frequencies observed in the study. The effect sizes can also shed light as to why there are some differences between the two countries, given that the effect sizes were different for each country and for each shape property. Scale for effect sizes is different in each sample.

Significant main effects for all three shape properties were found in both countries (see Figure 6 and Table 3). Rounder shapes were more easily matched with the word sweet than were more angular shapes (OR UK: 1.57; OR Colombia: 12.3). More symmetrical shapes (OR UK: 4.24; OR Colombia: 3.42) and shapes that had fewer elements (OR UK: 3.45; OR Colombia: 3.65) were also more likely to be matched to the word sweet when compared with asymmetrical shapes and those shapes having a greater number of elements, respectively. Overall, symmetry/asymmetry was most influential in terms of how shapes were matched to a specific taste word in the UK sample. For the Colombian sample, roundness/angularity was the shape property that had the greatest influence on shape-taste matches.

**Table 3:** **The influence of each shape property on shape-taste matches as shown by binary logistic regression GEE**.

**Shape properties**	**UK**	**Colombia**
Roundness/angularity	Angular = sour^**^	Angular = sour^***^
Symmetry/asymmetry	Symmetrical = sweet^***^	Symmetrical = sweet^**^
Number of elements	Fewer elements = sweet^***^	Fewer elements = sweet^***^
Roundness/angularity × symmetry	No effect	No effect
Roundness/angularity × number of elements	Rounder × fewer elements = sweet^**^	Rounder × fewer elements = sweet^***^
Symmetry/asymmetry × number of elements	Symmetrical × fewer elements = sweet^**^	No effect
Roundness/angularity × symmetry/asymmetry × number of elements	Rounder × symmetrical × fewer elements = sweet^*^	No effect

Note that all effects that are significant include both polar properties (e.g., symmetry and asymmetry, or roundness and angularity).

* p < 0.05;

** p < 0.01;

*** *p < 0.001*.

The congruent pairing of roundness/angularity and number of elements appeared to influence the speed of categorization in terms of the words sweet or sour in both countries (OR UK: 8.29; OR Colombia: 5.88, see also Table 3). For the UK sample, congruent symmetry/asymmetry × number of elements (OR: 1.73) and roundness/angularity × symmetry/asymmetry × number of elements combinations (OR: 3.29) increased the odds of matching a shape with a specific taste word (see Table 3).

### RTs

**Figure 7:**
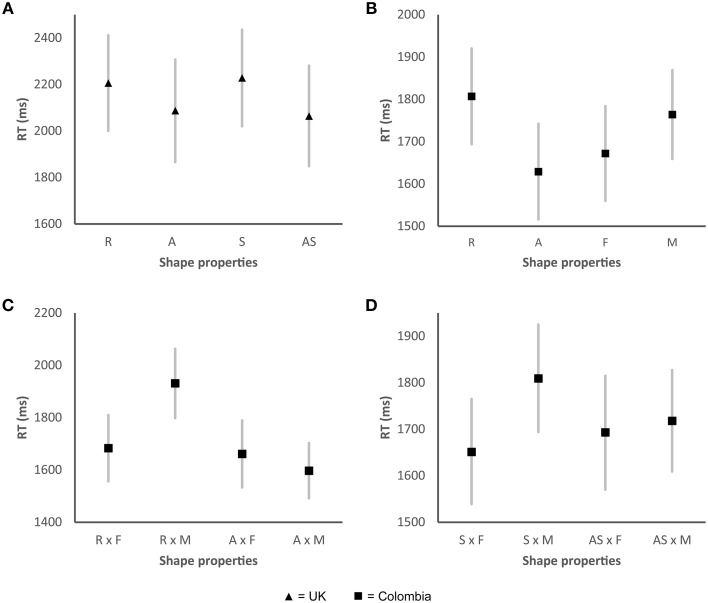
**Graphs showing the mean RTs for the taste task for those shape properties that displayed significant differences. (A)** Shape properties that displayed a significant main effect for the UK sample; **(B)** Shape properties that displayed a significant main effect in the Colombian sample; **(C)** Significant two-way interaction between roundness/angularity and the number of elements for the Colombian sample; **(D)** Significant two-way interaction between symmetry and the number of elements for the Colombian sample. R, round; A, angular; S, symmetrical; AS, asymmetrical; F, fewer elements; M, more elements. Error bars, standard error.

In the UK sample, significant main effects of roundness/angularity Wald $\chi_{\left(1\right)}^{2}=5.13$, *p* < 0.05 and symmetry/asymmetry Wald $\chi_{\left(1\right)}^{2}=10.93$, *p* < 0.01 were observed. Angular shapes (CI: 1866–2306 ms) were, on average, categorized 119 ms faster than rounder shapes (CI: 2000–2411 ms). The participants responded 164 ms more rapidly to the asymmetrical shapes (CI: 1848–2281 ms) than to the symmetrical shapes (CI: 2020–2435 ms, see also Figure 7A).

The Colombian participants displayed significant RT differences for roundness/angularity Wald $\chi_{\left(1\right)}^{2}=13.77$, *p* < 0.001, as well as for the number of elements, Wald $\chi_{\left(1\right)}^{2}=6.76$, *p* < 0.01 (see Figure 7B). Angular shapes (CI: 1538–1794 ms) were categorized 210 ms (*p* < 0.001) faster than rounder shapes (CI: 1743–2010 ms). Shapes with fewer elements (1602–1857 ms), yielded faster responses (83 ms, *p* < 0.05) than those shapes with more elements (CI: 1692–1934). Significant interactions were found between the number of elements possessed by a shape and both roundness/angularity Wald $\chi_{\left(1\right)}^{2}=17.17$, *p* < 0.001 (see Figure 7C) and symmetry/asymmetry Wald $\chi_{\left(1\right)}^{2}=4.35$, *p* < 0.05 (see Figure 7D). Those shapes that were both angular and had a greater number of elements (CI: 1567–1843 ms) presented the slowest RTs (1705 ms), whereas shapes with more elements and that were also round produced the slowest RTs (1999 ms). Shapes that were both asymmetrical and presented fewer elements were categorized more rapidly (1727 ms) than shapes with other combinations of the same two polar properties (i.e., roundness/angularity and number of elements). Participants' produced the slowest RTs when asked to categorize those shapes with a greater number of elements and that were also symmetrical (1865 ms), as compared with shapes with varying arrangements of the same two polar properties (i.e., symmetry/asymmetry and number of elements).

### Motor complexity

**Figure 8:**
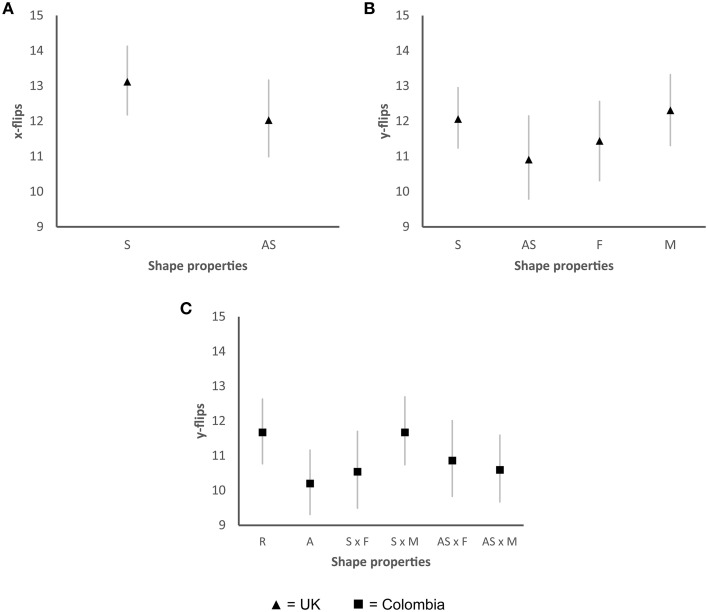
**Graphs showing the mean hand flips for the taste task for those shape properties that displayed significant differences. (A)** Significant differences between symmetry and asymmetry for x-flips in the UK sample; **(B)** Significant differences between symmetry and asymmetry, and between the number of elements for y-flips in the UK sample; **(C)** Significant differences between roundness and angularity and significant interaction between symmetry/asymmetry and the number of elements for y-flips in the Colombian sample. Significant differences were found in the Colombian sample when comparing shapes that were both asymmetrical and had fewer elements with those shapes that were symmetrical and had more elements (S × F vs. S × M). Significant differences were also found when comparing symmetrical and shapes with more elements with asymmetrical shapes with the same number of elements (S × M vs. AS × M). S, symmetrical; AS, asymmetrical; F, fewer elements; M, more elements. Error bars, standard error.

In the taste task, a significant main effect for symmetry/asymmetry was found for the UK sample for hand flips in both the x-axis Wald $\chi_{\left(1\right)}^{2}=10.06$, *p* < 0.01 (see Figure 8A) and the y-axis Wald $\chi_{\left(1\right)}^{2}=7.61$, *p* < 0.01 (Figure 8B). The number of elements present in a shape also displayed a significant main effect on the number of y-flips that were generated by the participants while categorizing each shape Wald $\chi_{\left(1\right)}^{2}=4.09$, *p* < 0.05 (see Figure 8B). In the Colombian sample, roundness/angularity displayed a significant main effect for hand flips in the y-axis Wald $\chi_{\left(1\right)}^{2}=11.21$, *p* < 0.001 (see Figure 8C). A significant interaction between symmetry/asymmetry and the number of elements was also found for hand flips in the y-axis Wald $\chi_{\left(1\right)}^{2}=5.30$, *p* < 0.05 (as also shown in Figure 8C). Overall, the participants generated more hand flips for those shapes that were more symmetrical shapes and had a greater number of elements. Specifically for the Colombian sample, rounder shapes also generated more hand flips. No other effects were found.

### Task comparison

In order to compare whether the participants exhibited a tendency to match the same shapes to sweetness and pleasantness and to sourness and unpleasantness, the responses from the UK and Colombian samples were aggregated (8 shapes × 63 participants = 504). Since each participant categorized each shape four times on each task, the categorizations were transformed into ranked values depending on how many times a shape was consistently classified as sweet/pleasant or sour/unpleasant (i.e., 4 = all were consistently associated with the same word; 3 = ¾ of times a specific shape was associated with the same word; 2 = half of the times a shape was associated with one of the words; 1 = a shape was associated with that particular word only once, also see Table 4). This is the reason why in this particular instance we only have 504 data points instead of the original 2016 trials. As a first step, a Pearson correlation was computed to assess the relationship between the responses in the valence and taste task. A moderate significant correlation was found for the matches made by the participants in the two tasks (*r* = 0.42, *n* = 504, *p* < 0.01); showing that those participants who matched a shape with the word pleasant also associated the same shape with the word sweet. The correlation also revealed that the participants tended to match those shapes with sourness that were also associated with the word unpleasant. Furthermore, the choices made by the participants were classified in terms of their consistency; that is, whether the participants matched a shape to the same word across the four trials. After this process had been completed for both tasks, standardized residuals were calculated to determine if the participants categorized shapes as pleasant-sweet and as unpleasant-sour. The standardized residuals (SR, see Table 4) show that shapes that were matched to the word sweet were also more likely to be matched to the word pleasant. Moreover, shapes that were categorized as sour were also more likely to be matched to the word unpleasant. Shapes that were matched to the word sour or unpleasant were less likely to be matched to the word sweet or pleasant and vice-versa.

**Table 4:** **Frequencies and standardized residuals used to compare responses between tasks**.

**Taste/Valence**		**Pleasant 4/4**	**Pleasant 3/4**	**50–50 2/4**	**Unpleasant 3/4**	**Unpleasant 4/4**	**Total frequency**
Sweet 4/4	Frequency	124	33	15	29	47	248
	SR	3.5	1.5	−1.1	−0.4	−3.8	
Sweet 3/4	Frequency	16	8	2	2	9	37
	SR	0.7	2.1	−0.5	−1.2	−0.9	
50–50 2/4	Frequency	16	3	4	4	5	32
	SR	1.3	−0.2	0.9	0.0	−1.7	
Sour 3/4	Frequency	10	0	3	6	13	32
	SR	−0.5	−1.8	0.3	1.0	0.8	
Sour 4/4	Frequency	18	8	16	23	90	155
	SR	−5.1	−2.0	1.1	0.7	5.6	
Total frequency		184	52	40	64	164	504

*Larger standardized residuals suggest a greater tendency to match a shape in both tasks consistently (i.e., pleasant and sweet, or unpleasant and sour)*.

*Fractions on each row and column represent the number of trials that were consistently matched with a specific word. SR = Standardized residuals*.

The results also revealed that symmetry/asymmetry influenced how easy it was for participants to match a shape to both the word pleasant and the word sweet (*SR* = 2.4). On the other hand, shapes that were more asymmetrical were more consistently matched by the participants with the words unpleasant and sour (*SR* = 2). The number of elements that make-up each shape also had an influence on how likely it was that shapes were categorized as both pleasant and sweet (*SR* = 2.9) and unpleasant and sour (*SR* = 1.4).

## Discussion

The present study evaluated how roundness/angularity, symmetry/asymmetry, and the number of elements influenced participants' categorization judgments concerning the eight different shapes in the two tasks. In one task, the participants had to categorize the shapes as either pleasant or unpleasant, while in the other task, they had to categorize them as sweet or sour instead. The results demonstrated that roundness/angularity, symmetry/asymmetry, and the number of elements exerted different degrees of influence over the likelihood that a given shape would be matched to a specific valence and taste word. The results reported here demonstrate that symmetry/asymmetry and the number of elements can influence the perceived valence of a shape; they also reveal that roundness/angularity requires that symmetry/asymmetry be present in order to convey a specific valence. Regarding shape-taste matches (second task), the results revealed that all three shape attributes (i.e., roundness/angularity, symmetry/asymmetry, and the number of elements) exerted varying degrees of an influence over the way in which shapes are matched to the words sweet and sour. It would seem that symmetry/asymmetry is an important property when it comes to conveying sweetness/sourness. What is more, since, by itself, roundness did not present a significant main effect in terms of valence, if affective matching actually mediates shape-taste correspondences, shape attributes such as symmetry/asymmetry may have a significant contribution when matching shapes to sweet and sour tastes.

The fact that we did not find an effect for roundness may be explained by the type of task used. Previous studies primarily used scales to measure preferences for roundness (e.g., Bar and Neta, 2006), whereas here we presented the participants with a forced choice that is perhaps less sensitive to effects that may be smaller. Differences in the sensitivity of ratings using scales and a forced choice task are mainly based on the fact that the forced choice task offers no opportunity for the participant to grade their response. However, the inclusion of mouse tracking offers the possibility of being able to measure participants' hesitations and association strength (Yu et al., 2012), thus providing an interesting and novel tool with which to study crossmodal correspondences. In this sense, our study does not contradict previous studies that have demonstrated that roundness is indeed a key property that can be associated with a specific valence or taste. Rather, our results highlight the importance of symmetry/asymmetry and the number of elements on shape-valence and shape-taste matches. The results of the present study, also suggest that given the influence that these shape attribute had on the odds of matching a shape to either a valence or a taste word, it is important to take different attributes into account in research interested in shape-taste correspondences.

Comparing the responses in the two tasks, the correlations and standardized residuals (see Table 4) show that there is some consistency in the matches made by the participants across tasks. Hence, our results support the view that emotional valence is involved shape-taste matching. However, given that the correlation and residuals did not present a large effect, this underscores the existence of other factors that mediate shape-taste correspondences too. More research is therefore needed to understand the mechanisms that facilitate the bidirectional matching of shapes and tastes.

Regarding the RT data, the participants responded more quickly to stimuli that were matched to a negative valence (i.e., unpleasant). Analysis of the RT data also revealed that shapes that presented properties that had a congruent valence (e.g., shapes that were both symmetrical and presented fewer elements) produced faster responses than those shapes that had an incongruent underlying valence (e.g., symmetry and a greater number of elements). This effect was further enhanced for those shapes that not only presented congruent shape properties but that were also matched to a negative valence. This is consistent with evidence showing that unpleasant stimuli tend to elicit faster RTs (e.g., Boesveldt et al., 2010). What is more, processing fluency (Reber et al., 2004) offers a plausible mechanism with which to explain why shapes that present properties with a congruent valence yield faster RTs.

Furthermore, motor complexity (that is, hand movements prior to clicking the mouse) may provide an interesting candidate with which to evaluate association strength between a stimulus and the target response. Moreover, hand movements could also indicate when a stimulus is harder to process or interpret. For example, those shapes that are made-up of properties that communicate contradictory opposite valences or different tastes (e.g., sweet or sour) may produce more hand movements. This suggestion is supported by the results of the present study showing that increased motor complexity indexes for those shapes presenting mismatched shape properties (e.g., symmetry and a greater number of elements, see Figures 5, 8). What is more, hand movements could help shed light on the factors that may contribute to RT latencies and on the underlying behaviors that accompany them. So, for example, one thing is to find that there are fewer hand movements and a slower RT, than a similar RT with a greater number of hand movements. The first might suggest a longer time to accumulate information to make a decision, whereas the latter could point to competing or weak associations between the stimulus and the possible target responses (see Spivey and Dale, 2006; Barca and Pezzulo, 2015).

Looking at RTs and motor complexity indexes together, it is possible to observe that our participants found it easier to determine when a shape was unpleasant or sour, than when it was pleasant or sweet. This can be seen both in the RTs and flips of those shapes that were categorized as unpleasant or sour. Faster RTs and less hand flips may relate to higher processing fluency (Reber et al., 2004) as well as stronger implicit associations (Yu et al., 2012) between a shape and the words unpleasant and sour. Our results are thus consistent with Bertamini et al.'s (2015) recent suggestion that the preference for roundness could also be mediated (to a degree) by a dislike for angularity. Bertamini et al.'s findings could also be extended to other shape properties, as suggested by the results of the present study.

Our results, showing that symmetry had a strong influence over the matching of shapes to valence and taste words, is consistent with evidence demonstrating that symmetry is a positive salient feature (Jacobsen et al., 2006). Indeed, throughout evolution, symmetry has been used as a signal of biological fitness, resources, and overall quality (Enquist and Arak, 1994). It is likely that the pervasive presence of symmetry across different contexts and situations that are associated with an advantage to the organism ultimately means that this visual property is always going to be associated with a positive valence. From there on, it would not be difficult to match other stimuli that are associated with a positive valence to symmetry (e.g., sweetness; see Yarmolinsky et al., 2009). Regarding the number of elements, Gordon and Holyoak (1983) reported that the liking for complex visual stimuli was inversely correlated to the distortion of the stimuli. What is more, there is evidence that symmetry has a moderating influence on the number of elements; specifically reducing the perceived complexity of a shape (see Tinio and Leder, 2009). Asymmetry may also have an influence on how the number of elements in a shape are perceived. This seems to be supported by our results, given that shapes that were both asymmetrical and presented a greater number of elements presented the most consistent matches with the word unpleasant.

With reference to the RT data, those shapes that were either incongruent (e.g., round, asymmetrical, and presented more elements) and also those shapes that presented more elements tended to yield faster RTs (especially when asymmetry was present). There are two potential, and not necessarily exclusive, explanations for such results. On the one hand, there is evidence to suggest that complex shapes can actually be processed more rapidly than simple shapes, because they have more redundant information (Biederman et al., 1991). Nevertheless, those shapes with properties that communicate opposite valences should still be more difficult to process than those shapes that consistently present the same valence. This is supported by our motor complexity results which show that the participants generated more hand movements, prior to clicking the mouse, when presented with incongruent shape properties. By incongruent, we refer to those shapes that displayed properties with contradictory valence/taste associations, for example symmetry (pleasant/sweet) and a greater number of elements (unpleasant/sour). Motor complexity may suggest that participants had a harder time classifying a particular shape. On the other hand, there is evidence to suggest that conflicting sensory information can be construed as an aversive signal by the brain (Dreisbach and Fischer, 2012; see also Treisman, 1977). Consequently, the fast RTs generated by participants when classifying the shape that was rounder, asymmetrical, and presented more elements, in the valence task, could be due to its incongruence (i.e., some properties may be communicating a positive valence while other a negative valence). The incongruence between the elements could be construed by the brain as conflicting information and thus generate an aversive signal that yields faster RTs.

The effect size differences found for each shape property between the UK and Colombian participants are consistent with evidence showing that there are some individual and cultural variations for the preference of visual properties and the arrangement of those properties (e.g., Masuda et al., 2008; Tinio and Leder, 2009; see also Jacobsen, 2010, for a review of cultural and individual differences in aesthetic preferences). The evidence presented here contributes to further understanding how different visual properties are perceived (and judged) in different countries, as well as how these properties influence behavior. The fact that symmetry/asymmetry, had such a stable influence over both tasks and measures suggest that certain properties present a more stable effect across participants. This result underscores the importance of understanding how different shape properties contribute to the matching of shapes to tastes. For example, while our results are consistent with previous findings regarding the matching of shapes and tastes, they also show that different shape properties influence this matching in each country.

The results of the valence task in the UK participants revealed that the overall effect of symmetry/asymmetry was six times greater than that of the number of elements present in the shape. By contrast, the effect of symmetry/asymmetry was a little under three times greater than that of the number of elements in the Colombian sample. This result suggests that symmetry/asymmetry had a dominating effect on how shapes were matched to a specific valence in UK sample, whereas there was a shared effect of symmetry/asymmetry and the number of elements (with symmetry still having the greatest overall influence) on shape valence-matches in the Colombian sample. In the taste task, the relevance of taking into account different shape properties in the study of shape-taste correspondences becomes even clearer. In the UK sample symmetry/asymmetry and the number of elements displayed a similar effect size (with roundness/angularity following close) over how shapes are matched to a specific taste. On the other hand, roundness/angularity had a dominating influence in the Colombian sample, with symmetry/asymmetry and the number of elements having a much smaller effect. This study provides preliminary evidence showing that the valence hypothesis to explain shape-taste correspondences maybe incomplete. Furthermore, we found that symmetry/asymmetry presented the greatest effect size in the valence task. On the other hand, symmetry/asymmetry, and the number of elements, both played key roles in shape-taste matches in the UK sample, while roundness/angularity presented the highest effect in the Colombian sample. Nevertheless, shape-taste matches were similar in both countries, thus suggesting that there may be other mechanisms that were not included in our study. Further research is therefore needed in order to determine what specific properties facilitate the matching of shapes and tastes to a specific valence (and to each other). Our results suggest that somewhat different shape properties facilitate shape-taste matches in each country.

Tinio and Leder's (2009) study revealed that preferences toward different visual properties were mediated by familiarity. That said, responses to those features that are based on preferences may not have the same influence across different cultures. As Tinio and Leder suggest, those individuals who are used to viewing objects that present fewer elements tend to like objects that present a greater number of elements. Moreover, Weierich et al. (2010) have demonstrated that novelty is a property with inherent affective value. It is also known to increase arousal. Bearing this in mind, perhaps it is novelty rather than familiarity which can influence the valence that is attributed to different shape properties. It would be interesting to take into account novelty as a variable in future studies and determine whether it can influence the effect and effect sizes of different shape properties (e.g., symmetry/asymmetry and the number of elements). In addition, another potential (complementary) explanation for the cultural differences reported in the present study relates to sound symbolism. Previous studies have shown that speech sounds are also involved in crossmodal correspondences (e.g., Spence and Gallace, 2011; cf. Simner et al., 2010). It is therefore just possible that the phonetic differences between the English and Spanish pronunciations of the words sweet/dulce, sour/ácido, pleasant/agradable, and unpleasant/desagradable might just possibly have influenced the matches of specific shape attributes to a taste or a valence word. What is more, because language is adapted in everyday life and it is also possible that the arousal or overall valence of each these words may not be the same in each language (or culture). As far as we are aware, to date, there are no studies that have probed whether the valence or arousal level generated by a word can influence crossmodal correspondences. However, it is important to note that previous studies have found similar results for taste words and actual tastants (Velasco et al., 2015a). The aforementioned findings are complemented by a study conducted by Ngo et al. (2013) in which they asked participants from the UK and Colombia to taste different fruit juices and rate them in terms of sound and shape symbolism scales. The study demonstrated that indeed participants from both countries matched the fruit juices in a similar manner and that shape and sounds symbolism scales yielded similar findings. Accordingly, the results of the present study seem to extend beyond a linguistic/semantic effect and do suggest that factors such as valence and aesthetics can influence crossmodal matching.

Finally, the findings of the present study may potentially have implications for the fields of packaging and label/logo design since they offer guidelines when it comes to matching visual information to specific tastes. It would be interesting to conduct further research with a greater variety of stimuli, given that we used just one shape for each condition. Nevertheless, the clear effect reported for symmetry on both samples is consistent with previous findings on aesthetics and evolutionary biology showing that symmetry is preferred over asymmetry. Our results are also consistent with findings demonstrating that roundness is more easily matched with sweetness and angularity to sourness.

The effects reported in the present study could be explained via different mechanisms. On the one hand it is possible that semantic associations in both Spanish and English regarding the word sweet and sour with both a taste and a valence could influence the matches made by the participants. On the other hand, there is a growing body of research showing the pervasive and structural influence of visual properties without any sort of context and even when presented for a few seconds. For example, angular shapes seem to active neural circuitry for threat detection (e.g., Larson et al., 2009, 2012), while symmetry is a preferred quality across species and contexts (e.g., Enquist and Arak, 1994; Sasaki et al., 2005). The notion that the aforementioned properties inherently communicate a valence (or a taste) still requires further study (especially given the forced-choice nature of the present study). It is important to continue to establish the existence of robust correspondences between shapes and tastes (and between properties in other sensory modalities). Nevertheless, it is also important to conduct studies that go beyond behavioral measures and that can help determine if there are distinct patterns of neural activation for the different kinds of crossmodal correspondences (see Spence and Parise, 2012; Spence and Deroy, 2013b; Parise, 2015).

The findings of our study further contribute to the development of design guidelines for brands, products, and advertising. There is already evidence to show that the congruency between different sensory attributes (e.g., how round the shapes present in a brand logo are, or the match between the shape of the packaging and the taste of the product) can have a positive influence on the emotional judgments made by consumers (Salgado-Montejo et al., 2014). Furthermore, the present study could help us to understand how different visual properties can be associated with a specific valence and taste and hence have an impact on the expectations and experiences that people can have when interacting with different elements in the real world. Understanding how visual information can convey emotion and taste is of interest to designers, marketers, brand owners, and those entrepreneurs seeking to convey experiences associated with a specific taste and/or valence. Finally, the ever-growing interest (and pressure) to innovate and develop new and more attractive products may overlook important health and safety practices. Specifically, there have been reported cases of accidental poisoning caused by consuming “food imitating products,” that is, products from a non-food category that have features that make them resemble a food product (Basso et al., 2014).

In future studies, it will be interesting to include novelty and familiarity as covariates in order to determine their influence on shape-taste matches. Furthermore, it would be interesting to determine if the effects reported here can be extrapolated to more complex stimuli (e.g., products, brands, and websites, see Crilly et al., 2004; Lavie and Tractinsky, 2004; Salgado-Montejo et al., 2014; Velasco et al., 2014). Moreover, given the key role played by symmetry/asymmetry in both shape-valence and shape-taste matches, it would also be interesting in future research to investigate whether auditory symmetry/asymmetry (Kempf, 1996; Dean et al., 2009) might also be matched to sweet/sour tastes, respectively. To the best of our knowledge, the present study is the first to consider symmetry and number of elements as a possible visual attributes that can facilitate shape-taste matches.

### Conflict of interest statement

The authors declare that the research was conducted in the absence of any commercial or financial relationships that could be construed as a potential conflict of interest.

## Footnotes

^1^Taste here is used to mean the sensation that arises from the stimulation of gustatory receptors on the tongue rather than the broader concept of flavor which also includes olfactory and trigeminal information (see Spence et al., 2015).

^2^GEE is a quasi-likelihood method that extends from generalized linear modeling. Aside from the benefit of controlling for multiple responses, it can also control for data that is not independent (which is the case in many repeated measure designs). While a logistic regression could have been used to analyze the data, the risk of a Type-II error is higher than with a GEE, since it does not properly control for repeated as well as for dependent measures (increasing variance redundancy; see Hanley et al., 2003, for a discussion on the suitability of GEE).

## Appendix

**Table A1:** **Statistical analyses carried-out for the present study by task, country, measure, and factor**.

**Measure**		**Task**							
		**Valence**				**Taste**			
		**χ2**		***p***		**χ2**		***p***	
		**UK**	**Col**	**UK**	**Col**	**UK**	**Col**	**UK**	**Col**
**CATEGORIZATION**									
	RA	2.39	0.60	0.12	0.44	4.97	52.45	*p* < 0.05	*p* < 0.001
	SAS	54.51	33.07	*p* < 0.001	*p* < 0.001	13.85	11.86	*p* < 0.001	*p* < 0.001
	NE	25.01	37.26	*p* < 0.001	*p* < 0.001	22.41	36.16	*p* < 0.001	*p* < 0.001
	RA × SAS	4.63	0.42	*p* < 0.05	0.52	0.67	0.19	0.41	0.67
	RA × NE	0.56	7.74	0.46	*p* < 0.01	8.32	17.95	*p* < 0.01	*p* < 0.001
	SAS × NE	11.68	18.43	*p* < 0.001	*p* < 0.001	8.90	3.13	*p* < 0.01	0.08
	RA × SAS × NE	1.50	10.19	0.22	*p* < 0.001	3.89	0.16	*p* < 0.05	0.69
**RT**									
	RA	0.32	1.92	0.57	0.17	5.13	13.77	*p* < 0.05	*p* < 0.001
	SAS	8.45	2.06	*p* < 0.01	0.15	10.93	1.12	*p* < 0.001	0.29
	NE	15.88	0.85	*p* < 0.001	0.36	0.08	6.76	0.78	*p* < 0.01
	RA × SAS	0.01	1.90	0.92	0.17	2.74	1.14	0.10	0.29
	RA × NE	1.01	0.60	0.32	0.44	0.58	17.17	0.45	*p* < 0.001
	SAS × NE	8.66	12.45	*p* < 0.01	*p* < 0.001	2.72	4.34	0.10	*p* < 0.05
	RA × SAS × NE	6.33	<.001	*p* < 0.05	0.97	0.61	0.91	0.43	0.34
**X-FLIPS**									
	RA	0.25	0.47	0.62	0.49	2.01	3.34	0.16	0.07
	SAS	1.30	1.62	0.25	0.20	10.06	0.29	*p* < 0.01	0.59
	NE	0.19	0.14	0.67	0.71	0.33	0.44	0.57	0.51
	RA × SAS	0.02	0.72	0.88	0.40	3.20	0.07	0.07	0.80
	RA × NE	0.72	0.01	0.40	0.91	0.02	3.27	0.90	0.07
	SAS × NE	4.92	3.76	*p* < 0.05	0.05	0.74	0.25	0.39	0.62
	RA × SAS × NE	1.35	0.13	0.25	0.71	0.39	1.30	0.53	0.26
**Y-FLIPS**									
	RA	0.69	0.25	0.41	0.62	1.35	11.21	0.25	*p* < 0.001
	SAS	2.35	1.30	0.13	0.25	4.09	0.88	*p* < 0.01	0.35
	NE	10.26	0.19	*p* < 0.001	0.67	4.09	1.07	*p* < 0.05	0.30
	RA × SAS	0.52	0.02	0.47	0.88	3.56	<.001	0.06	0.99
	RA × NE	0.07	0.72	0.79	0.40	0.71	1.73	0.40	0.19
	SAS × NE	4.28	4.92	*p* < 0.05	*p* < 0.05	1.15	5.30	0.28	*p* < 0.05
	RA × SAS × NE	0.02	1.35	0.88	0.25	0.61	0.31	0.43	0.58

*Shading indicates significant effects or interactions*.
